# Editorial: Creative digital design and manufacturing in medicine

**DOI:** 10.3389/fbioe.2022.983701

**Published:** 2022-08-08

**Authors:** Mika Salmi, Jan Wolff, Antti Mäkitie

**Affiliations:** ^1^ Department of Mechanical Engineering, Aalto University, Espoo, Finland; ^2^ Department of Dentistry and Oral Health—Section of Oral and Maxillofacial Surgery and Oral Pathology, Aarhus University, Aarhus, Denmark; ^3^ Department of Otorhinolaryngology—Head and Neck Surgery, University of Helsinki and Helsinki University Hospital, Helsinki, Finland

**Keywords:** additive manufacturing, 3D printing, 3D medical imaging, 3d modeling and 3d models, medical models, biomaterials

Digitalization continues to take significant developmental steps in medicine. Simultaneously methods for digital design including 3D modeling and optimization and digital manufacturing methods such as additive manufacturing have improved significantly over the past decade. These processes offer clinicians the unique possibility of using existing and novel biomaterials, which rapidly open new medical applications. By combining engineering and medical skills with these technical developments, we can take an advanced leap in developing and studying new personalized medical treatments and devices with novel material combinations. These developments do however require novel thinking and creative approaches to be successful.

In clinical settings, typically, the first question asked concerning 3D printed constructs is related to the used material and its properties. In this context Chen et al. studied the degradation of 3D-printed porous polylactic acid (PLA) scaffolds under the mechanical stimulus. There were 45 samples printed and the degradation was assessed within 90 days. The results demonstrated that mechanical stimulus accelerated the degradation of PLA scaffolds. More specifically higher mechanical stimulus led to faster degradation of the scaffolds at the late stage of the degradation process. These findings could help in designing optimal biodegradable PLA scaffolds. In another study Fang et al. explored bioceramic scaffolds for bone tissue engineering. More specifically the structural, degeneration, permeation, and physiological activity of 3D-printed bioceramic biocomposites with various conformations and work systems from the macro to nano range were studied. Furthermore, their impact on mechanical, degeneration, porosity and physiological activities were investigated. The study also summarized recent advances and proposed a new therapeutic strategy focused on the extension of conventional ceramics.

In medical imaging, phantoms are currently used to assess the accuracy and the quality of different imaging modalities. Such phantoms are to date not mass-produced and are rather expensive. In this context, the production of phantoms could profit from novel additive manufacturing technologies. Ma et al. investigated X-ray attenuation properties of additive manufacturing materials using computed tomography with a tube voltage of 70–140 kVp. The HU values of the printed polymers ranged from −266 to +1,804, and for vat photopolymerization from 16 to 178 depending on tube voltage. The results of this study open new avenues for designing and manufacturing tailored phantoms thereby mimicking a wide range of different anatomical structures.

In clinical settings, the integration of novel technologies and the development of corresponding clinical workflows remains a challenge. In this context Xu et al. developed a digital workflow to design and manufacture custom-made short implants using a wing retention device. The implants are unique since they can be fixed to the subperiosteal or alveolar bone. These precise and minimally invasive designed implants offer patients with severe vertical bone height insufficiency in the posterior maxillary region new solutions. In another case Kong et al. developed a novel method to evaluate 3D-printed titanium augments combined with tantalum trabecular cups for patients who underwent revision of total hip arthroplasty. The midterm outcomes were satisfactory, and changes in the Harris Hip Score and Oxford Hip Score suggested a significant improvement in hip function.

Matehuala-Morán et al. designed a video-laryngoscope for endotracheal intubation of adult patients during the COVID pandemic since the cases of patients requiring endotracheal intubation increased markedly during the pandemic. The estimated cost of the proposed video-laryngoscope is around $400, including a 4.5-inch screen, manufacturing process, transportation, and taxes. Compared to video-laryngoscopes available on the market, it has become an affordable option for low-resource healthcare units and developing countries.

This Research Topic of articles demonstrates new development in the field of medical device material, design, and manufacturing. Digital manufacturing and design is a rapidly growing research area in medicine. This Research Topic offers a sneak peek of the attractive emerging and developing field. We hope it can attract the attention of researchers from diverse disciplines. On behalf of the Editorial Board, we sincerely thank the authors and staff at Frontiers, without whom this Research Topic would not have been possible.
